# Comeliness and Comfort

**Published:** 1875-10

**Authors:** 


### COMELINESS AND COMFORT.

We do not see the excellent boots, shoes, and lasts, patented by Mr. Joel McComber, of 14 Union Square, on exhibition at the Institute. We are sorry for this, because among the 500,000 visitors attending the Fair, there would surely be some who would there examine these superior goods for the first time. Of those who examined, there would be many who would discover the true principle in the method employed, and would seek to know more concerning the great improvement. These would visit Mr. McComber at his store, and after due investigation would become converts to the rational plan. The persons thus converted would surely convert others—so contagious are grand ideas—and thus the influence would widen and extend. The glad tidings of a possible freedom from those distortions of the feet to which civilized man has for generations been subjected, would surely find new avenues to human hearts through such an exhibition as Mr. McComber is competent to make. Not for his sake simply, but for the sake of suffering humanity, do we regret that he did not place his excellent goods on exhibition. Not for his sake, we say, for no man needs the added publicity to be obtained from such a display, less than does Joel McComber. His store is filled from early morning till late at night with sensible folks, who, having tried his system, or having seen those who have, decline to be tortured longer.

Nobody needs to be told at this late day that the feet of human beings have been dreadfully tortured by the workers in leather, in all past time. The literature of the world contains allusions which clearly prove this. The structure of the human foot has been but imperfectly considered by that cruelest of saints, St. Crispin. Every child knows that new shoes are apt to be instruments of torture. There is the nursery rhyme to teach the fact:—

> There was an old man of Toulouse,
> Who purchased a new pair of shoes;
> When asked, "Are they pleasant?"
> He said, "Not at present,"
> This wretched old man of Toulouse.

But it needs no one to inform us of the misery induced by shoes which pinch, and cramp, and deform. This suffering is a part of every one's experience. The question is, can this deformity and wretchedness be surely obviated?

We are certain that it can. Nobody, perhaps, if we except Mr. McComber, has given more thought to this topic than ourselves. We have examined this subject of clothing for the feet with the view of securing comfort for ourselves, and in order that we might be more competent to judge of what is best for the rest of mankind. We have written a great deal on the subject, and have the satisfaction of knowing that multitudes have followed our advice with great satisfaction.

In past numbers of the Journal we have described McComber's improvements, and have explained the difference between his symmetrical lasts, made in imitation of the foot, and the absurd pieces of wood upon which other men construct apparel for the feet. We can not spare the space to go over that part of the subject here. All we seek now, is to arrest the attention of all who take some pride in being neat, and natural, and comfortable, long enough to induce them to call on Mr. McComber, or send to his place of business for his descriptive pamphlet. Especially do we desire to gain for him and his cause the ear of the father, the mother, and those who, having young children committed to their care, are willing to do whatsoever in them lies to discharge their whole duty by them, and to save the boys and girls, who will by and by be men and women, from the very serious torture and deformity which attends the senseless system of shoemaking in common use. We know that Mr. McComber's system accomplishes this. We have tested it in a large number of cases, and can speak positively to this point. We are convinced that comfort and elegance and great durability, are combined in no other system. As these goods are constructed with strict reference to the anatomy of the foot, they are delightfully easy from the first. As they do not have to be stretched and pulled out of their original form by the foot in the breaking-in process, they will wear fully twice as long, we think, as any of the ordinary kind. They are so constructed that the foot rests evenly on the sole. Thus, the upper-leather does not get pushed over, to impart a slouchy appearance to the boot or shoe. They are all that can be asked or desired, and we recommend them to all with the most entire confidence.

